# EEG-Based Seizure Prediction Using Hybrid DenseNet–ViT Network with Attention Fusion

**DOI:** 10.3390/brainsci14080839

**Published:** 2024-08-21

**Authors:** Shasha Yuan, Kuiting Yan, Shihan Wang, Jin-Xing Liu, Juan Wang

**Affiliations:** 1 School of Computer Science, Qufu Normal University, Rizhao 276826, China; ykttt@hotmail.com (K.Y.); 19710891605@163.com (S.W.); wangjuansdu@163.com (J.W.); 2 School of Health and Life Sciences, University of Health and Rehabilitation Sciences, Qingdao 266114, China; liujinxing@uor.edu.cn

**Keywords:** seizure prediction, STFT, hybrid model, DenseNet, vision transformer

## Abstract

Epilepsy seizure prediction is vital for enhancing the quality of life for individuals with epilepsy. In this study, we introduce a novel hybrid deep learning architecture, merging DenseNet and Vision Transformer (ViT) with an attention fusion layer for seizure prediction. DenseNet captures hierarchical features and ensures efficient parameter usage, while ViT offers self-attention mechanisms and global feature representation. The attention fusion layer effectively amalgamates features from both networks, guaranteeing the most relevant information is harnessed for seizure prediction. The raw EEG signals were preprocessed using the short-time Fourier transform (STFT) to implement time–frequency analysis and convert EEG signals into time–frequency matrices. Then, they were fed into the proposed hybrid DenseNet–ViT network model to achieve end-to-end seizure prediction. The CHB-MIT dataset, including data from 24 patients, was used for evaluation and the leave-one-out cross-validation method was utilized to evaluate the performance of the proposed model. Our results demonstrate superior performance in seizure prediction, exhibiting high accuracy and low redundancy, which suggests that combining DenseNet, ViT, and the attention mechanism can significantly enhance prediction capabilities and facilitate more precise therapeutic interventions.

## 1. Introduction

Epilepsy, a prevalent neurological condition marked by repeated and unforeseeable seizures, impacts more than 50 million individuals globally [1]. Epilepsy attacks are extremely uncertain and sudden, which seriously affects the physical and mental health of patients [2]. The accurate prediction of epileptic seizures is crucial for developing appropriate patient care strategies, optimizing treatment plans, and potentially even providing warnings prior to the onset of seizures. Nonetheless, forecasting the onset of seizures continues to be a considerable challenge owing to the intricate and diverse characteristics of epilepsy. An electroencephalogram (EEG) is an effective tool to measure non-stationary signals of brain electrical activity. A large amount of evidence shows that the use of EEGs to diagnose and predict seizures has been widely studied [3,4].

In the early years of seizure prediction research, various machine learning algorithms were employed to analyze EEG data and identify seizure-related patterns. Techniques such as support vector machines (SVM) [5], decision trees [6], k-nearest neighbors (k-NN) [7], and naive Bayes [8] were used in conjunction with handcrafted features, typically derived from signal processing techniques extracted from the EEG signals. These features often included time–domain, frequency–domain, and time–frequency representations, such as mean, variance [9], spectral power [10], wavelet coefficients [11], power spectrum density [12], and entropy [13]. While these approaches demonstrated some success, their performance was often limited by the choice of features and the inherent variability of EEG data.

In the past few years, substantial progress has been made in epileptic seizure prediction through the application of deep learning methods. Various architectures, including convolutional neural networks (CNNs) [14], long short-term memory (LSTM) networks [15], and transformers [16], have been employed to address the challenges associated with seizure prediction, particularly the inherent temporal dependencies in EEG data. Truong et al. proposed a method using STFT preprocessing and classification using CNN to verify the feasibility of the method in several datasets [17]. Moreover, LSTM has demonstrated proficiency in capturing dependencies across sequences, making it particularly suitable for time series data, including EEG signals. Tsiouris et al. provided a new approach to introduce LSTM for the first time in seizure prediction studies, taking advantage of deep learning techniques in processing time series data to improve prediction accuracy and utility [18].

CNNs are frequently utilized in seizure prediction tasks because of their capacity to recognize spatial–temporal patterns within EEG information. They can autonomously extract hierarchical characteristics from unprocessed data, overcoming the limitations of handcrafted feature extraction in traditional machine learning methods [19]. As a specific type of CNN architecture, DenseNet has gained popularity in this domain due to its unique densely connected layers that enable efficient feature reuse and reduce the risk of overfitting [20]. Due to its unique ability to reuse features at multiple levels, it has shown improved performance compared to other CNN architectures in various tasks. Jana et al. converted EEG signals into images for feature extraction and classification of inter-ictal and pre-ictal states using DenseNet [21]. Jibon et al. proposed a hybrid deep learning model called LGCN-DenseNet for automatic seizure detection, which demonstrated the effectiveness of the DenseNet network in EEG signal analysis by reducing the vanishing gradient problem and enhancing feature propagation [22].

Transformers, a popular deep learning architecture, was originally developed for natural language processing tasks, and then the Vision Transformer (ViT) was proposed as an adaptation of transformers for image classification problems, where the input image is treated as a sequence of fixed-size patches [23]. ViT has shown top tier results in numerous computer vision tasks and has been utilized in seizure prediction research to identify long-range dependencies and global context within EEG data based on self-attention mechanism in recent years. Bhattacharya et al. employed the STFT for extracting time–frequency characteristics and leveraged transformer networks for classification, signifying the inaugural use of transformers in seizure prediction investigations [24]. Zhang et al. introduced an approach in which the raw EEG signal was first filtered to extract pre-episode and inter-episode markers, and the processed data were classified using the ViT model [25]. Hussein et al. proposed the Multi-channel Vision Transformer (MViT) model for the automated and simultaneous learning of spatio–temporal–spectral EEG features [26]. Deng et al. introduced a novel hybrid visual transformer (HViT) architecture with data uncertainty learning for seizure prediction [27].

In addition, transfer learning has emerged as a powerful technique for seizure prediction, leveraging pre-trained models to overcome the challenges of limited labeled data in the epilepsy domain [28]. By fine-tuning models pre-trained on large-scale images or time series datasets, researchers can take advantage of the learned features and adapt them to the specific task of seizure prediction. Transfer learning has been successfully applied to various deep learning architectures, including CNNs, LSTMs, and transformers, to improve performance and reduce training time [29]. By leveraging the knowledge acquired from pre-training, transfer learning can lead to better feature extraction and more accurate seizure prediction models.

By combining the strengths of DenseNet and ViT, our study aims to develop an advanced deep learning model for seizure prediction, targeting improved prediction accuracy and decreased false alarm rates. The primary motivation of this study is to improve seizure prediction outcomes by leveraging the complementary feature extraction capabilities of DenseNet and ViT. DenseNet excels at capturing local spatial–temporal features from EEG signals, while ViT is adept at identifying long-range dependencies and global patterns [30]. Our proposed method fuses the features extracted by these two architectures using an attention mechanism, enabling the model to dynamically balance the significance of both feature groups. In this investigation, a unique DenseNet–ViT hybrid method is presented for seizure prediction. The main contribution of this study can be outlined in the following:

- Multi-model architecture with DenseNet and ViT: A hybrid model is proposed, combining the strengths of DenseNet and ViT architectures. Local features are captured by the DenseNet component, while global patterns are focused on by the ViT component. This combination allows for effective learning from the input data, resulting in improved seizure prediction performance;
- Attention-based feature fusion layer: An innovative attention-based feature fusion layer is included, dynamically weighing features extracted from DenseNet and ViT based on their relevance to the prediction task, resulting in a more robust and effective representation of seizure prediction;
- Pre-trained model transfer and optimized training strategy: The pre-trained DenseNet and ViT models are transferred to patient-specific models, which could enhance initial performance by utilizing their learned representations. Optimized training strategies include hyperparameter optimization with Optuna and early stopping. These techniques work together to improve the performance of the network.

The rest of the paper is structured as follows: Section 2 introduces the used EEG dataset and describes the proposed model in detail with preprocessing, hybrid model classification, and post-processing. The experimental results are summarized in Section 3 and the discussion is described in Section 4. Section 5 is the conclusion of this work.

## 2. Materials and Methodology

### 2.1. EEG Dataset

In this study, the CHB-MIT scalp EEG dataset was utilized for our seizure prediction task, which includes the continuous scalp EEG recordings from pediatric patients. This dataset includes 24 cases from 23 patients, with cases 01 and 21 derived from the same individual; case 24 has no specific information. The dataset adheres to the international 10–20 system for electrode placement, which ensures a standardized spatial arrangement for EEG recordings [31]. Compared with intracranial EEG, this dataset records scalp EEG with more noise, which makes seizure prediction more difficult.

The scalp EEG signals within the dataset were recorded at a 256 Hz sampling rate and a 16-bit resolution. Annotation files of the dataset reveal that channel configurations often differ among various recordings. To preserve consistency in the analysis, 18 fixed channels were chosen: FP1-F7, F7-T7, T7-P7, P7-O1, FP1-F3, F3-C3, C3-P3, P3-O1, FP2-F4, F4-C4, C4-P4, P4-O2, FP2-F8, F8-T8, T8-P8, P8-O2, FZ-CZ, and CZ-PZ. The main objective of identifying and predicting epileptic seizures simultaneously is to enable early intervention, so seizures occurring within a 30 min interval between two consecutive events are regarded as a single seizure. The details of the CHB-MIT dataset are shown in Table 1.

### 2.2. Algorithm Framework

**Figure 1:**
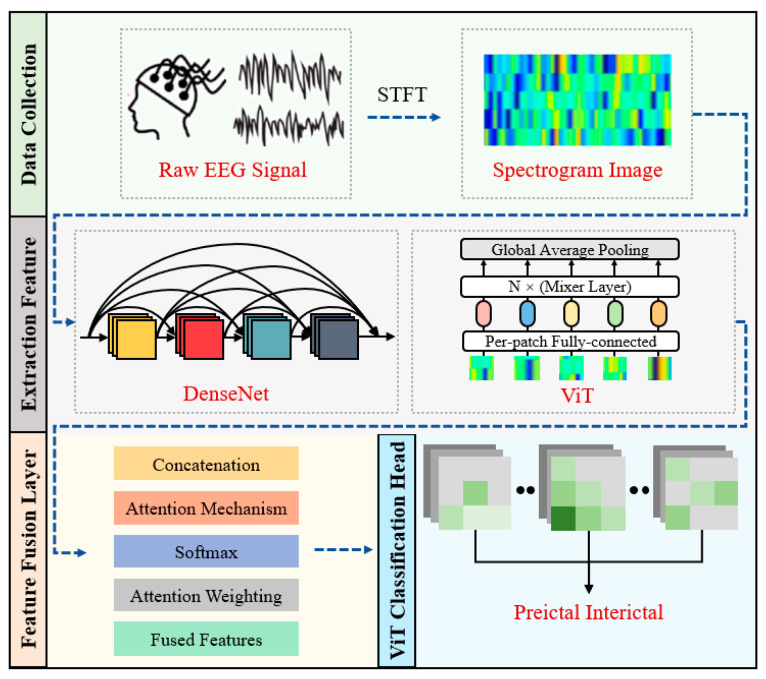
Overview of the proposed hybrid Densenet–ViT network model.

The proposed hybrid deep learning model for epileptic seizure prediction is shown in Figure 1. The raw EEG signal was firstly preprocessed using a 30 s short-time Fourier transform (STFT) to convert into a time–frequency matrix. This transformation could remove some EEG noise and allow the EEG data to be presented in a format that is more easily processed by following deep learning models. Subsequently, two pre-trained models, DenseNet and ViT, were employed to independently extract EEG features and were optimized to enhance their suitability for the seizure prediction task. Then, to effectively merge the strengths of DenseNet and ViT, an attention mechanism-based fusion layer was developed, which calculated the attention weights of the features extracted by each model, facilitating the joint consideration of local and global patterns of EEG data. Finally, after integrating the features of the two models, the original classification head of the ViT model was used to classify the merged features to obtain the final outputs and prediction results.

### 2.3. Preprocessing

The preprocessing was conducted on the raw EEG signals to facilitate analysis and enhance the model’s performance. Compared with other time–frequency analysis techniques, the STFT has the advantages of simple implementation and low computational complexity in EEG signal analysis. Hence, a STFT with a window length of 30 s was employed to transform the EEG signals into two-dimensional matrices, allowing for the extraction of crucial information in both time and frequency domains. For an EEG signal x(t), the STFT of it can be defined as

(1) $$STFT\{x(t)\}(f,\tau )=\int_{-\infty }^{+\infty }x(t)\cdot \omega (t-\tau )\cdot e^{-j2\pi ft}dt$$

where ω(t−τ) is the window function and f denotes frequency components.

In addition, the noise was addressed by eliminating power line noise in the 57–63 Hz and 117–123 Hz bands, as well as the DC component at 0 Hz. These measures were essential in reducing environmental interference and improving the quality of the input data. Furthermore, since the pre-ictal EEG data was less than the inter-ictal EEG data, this study used oversampling technology to increase the inter-ictal EEG data and solve the class imbalance problem, which could improve model performance.

### 2.4. Hybrid Model

**Figure 2:**
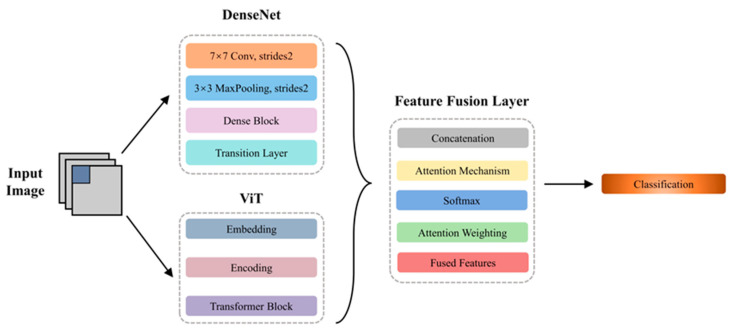
Structure of the hybrid DenseNet–ViT model, which contained a DenseNet module, ViT module, and feature fusion layer module.

In this study, an inventive hybrid model is proposed that capitalizes on the advantages of DenseNet and ViT for seizure prediction. The model aims to effectively capture both local and global features of the input EEG data by employing the strengths of DenseNet in handling spatial information and ViT in detecting long-range dependencies. The overall architecture comprised separate branches for DenseNet and ViT, followed by an attention-based fusion layer that intelligently combined the features extracted from both models. The resulting fused features were then inputted into the output layer for seizure prediction. As shown in Figure 2, the proposed hybrid DenseNet–ViT approach is presented.

#### 2.4.1. DenseNet

The DenseNet component of our hybrid model was designed to efficiently capture spatial patterns in the EEG data. DenseNet is a deep convolutional neural network architecture renowned for its resource efficiency and improved gradient flow [32,33]. Compared with traditional CNN, DenseNet can alleviate the gradient vanishing problem that occurs as the number of CNN layers increases. The input of each layer of the DenseNet network contains the output of the previous layers, which improves the feature transfer efficiency and network performance from the perspective of feature reuse. It is more suitable for training efficient feature information from a limited amount of EEG data. In addition, the connection of feature maps from the previous layer enables DenseNet to learn both low-level and high-level features, which helps to identify complex spatial patterns in EEG data.

Our implementation of DenseNet was derived from the DenseNet121 variant [34], encompassing 121 layers that included convolutional, batch normalization, and activation layers, all organized into dense blocks and transition layers. The dense blocks, the heart of the DenseNet architecture, contained multiple convolutional layers densely connected, facilitating feature reuse. For the output of the $i_{th}$ layer $X_{i}$,

(2) $$X_{i}=H_{i}([X_{0},X_{1},\ldots ,X_{i-1}])$$

where [•] represents the connection from layer 0 to layer i−1 and H represents nonlinear transformation. The $i_{th}$ layer receives the feature maps of all previous layers.

In contrast, transition layers help manage the spatial dimensions and channel depth of the feature maps as they propagate through the network. The original classification layer was removed and the output feature maps were used as the input to the attention-based fusion layer. This modified DenseNet architecture enables the extraction of meaningful spatial features from the EEG data, which is crucial for accurate seizure prediction.

#### 2.4.2. Vision Transformer

The ViT component of our hybrid model captured the long-range dependencies and global information in the EEG data. ViT employs the transformer technique and considers the input image as a series of fixed-size segments, transforming the two-dimensional image into a one-dimensional sequence of flattened patch embeddings [35]. Although RNN structures, such as LSTM networks, can also obtain the contextual information of EEG signals, the multi-head self-attention mechanism of ViT enables the model to establish long-range dependencies between different positions, thereby capturing global information, which is crucial for EEG signal analysis with a temporal nature and seizure prediction.

In this model, a pre-trained ViT-base-patch16-224 architecture was employed, which divided the input image into non-overlapping 16 × 16 patches and projected them into a 768-dimensional embedding space. The model also incorporated positional encoding to maintain spatial information. The inputs were then processed by a series of multi-head self-attention and feed-forward layers, forming the transformer blocks. The multi-head self-attention mechanism calculation can be expressed as

(3) $$MultiHead(Q,K,V)=Concat(head_{1},\ldots ,head_{h})W^{O}$$

(4) $$head_{i}\;=Attention(QW_{i}^{Q}\;,KW_{i}^{K}\;,VW_{i}^{V})$$

where Attention(Q,K,V) is the self-attention function and $W_{i}^{Q}\;,W_{i}^{K}\;,W_{i}^{V}$ are weight matrixes corresponding to query, keys, and values of each head.

The ViT component effectively captured the temporal relationships within the EEG data. When combined with the spatial features extracted by the DenseNet component, an effective seizure prediction model can be obtained.

#### 2.4.3. Feature Fusion Layer

In the proposed hybrid DenseNet–ViT model, a key innovation was the attention mechanism-based feature fusion layer. Its purpose was to effectively combine the fine-grained spatial feature extraction capabilities of DenseNet with the global contextual capturing advantages of ViT. This achieved a fusion of DenseNet’s local receptive fields and ViT’s global receptive fields, more comprehensively capturing the features of images. Consequently, this enhanced the accuracy of epilepsy seizure predictions using EEG data. The feature fusion layer ingeniously leveraged the attention mechanism, integrating the most representative features across spatial and temporal dimensions to construct a comprehensive and robust feature representation.

**Figure 3:**
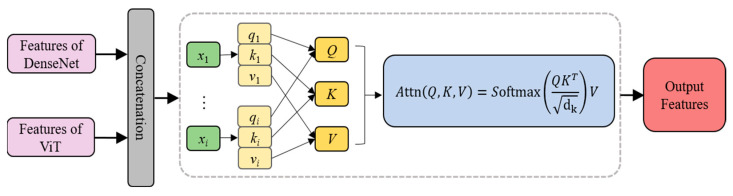
Structure of feature fusion layer with attention mechanism.

The feature fusion module based on the attention mechanism is shown in Figure 3. Firstly, the feature outputs of DenseNet and the sequence embeddings of ViT were input to the fusion layer in parallel. These inputs were merged along the channel dimension to form a unified and comprehensive feature representation. Then, the attention scores were calculated using the input features (as values) and associated queries and keys. These scores were normalized by a softmax layer to ensure their sum equaled one, indicating the relative importance of each feature. After that, these attention scores were used to weight the corresponding features, thereby amplifying those features more influential for the prediction task while diminishing the impact of less important features. Finally, the weighted features were fed into the classifier for the final prediction.

### 2.5. Postprocessing

During the inter-ictal phase, sporadic false positives might lead to unwarranted alerts. To tackle this problem, a post-processing stage was incorporated, utilizing the k-of-n strategy [17]. This approach minimized the false alarm rate by necessitating a specific amount of successive positive predictions prior to activating an alert. In the experiment, the values of n and k were set to 10 and 8, respectively, signifying that a minimum of 8 out of 10 continuous EEG signal segments must be classified as positive before activating the alert.

## 3. Experimental Section

### 3.1. Experimental Details

The experiment was conducted on a high-performance workstation equipped with an i7-12700K CPU of Intel and 128 GB of RAM of Kingston, operating under Windows 10 OS. During the model training process, a GeForce RTX 3080Ti GPU of NVIDIA was utilized, with acceleration enabled by CUDA technology. The development environment was established using Python 3.7 and PyTorch 1.10.

In this experiment, the Optuna hyperparameter optimization framework was applied, which used an advanced algorithm based on Bayesian optimization, and could find the optimal parameters faster than a traditional grid search or random search. Moreover, the Adam optimizer was combined with the cosine annealing strategy to help the model dynamically adjust the learning rate and avoid falling into the local minimum, which could improve the generalization ability of the model and better convergence.

### 3.2. Evaluation Approach and Results

**Figure 4:**
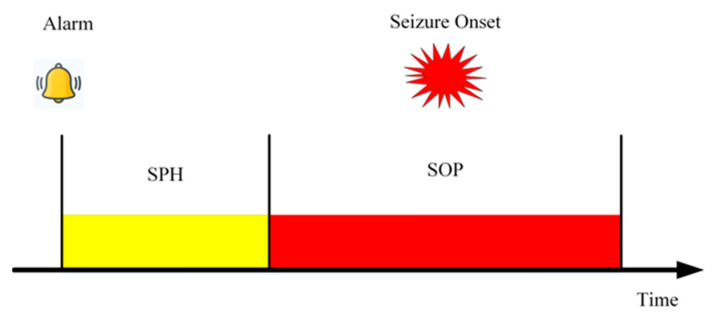
Schematic representation of seizure occurrence period (SOP) and seizure prediction horizon (SPH).

This study adheres to the standards proposed by Maiwald et al. [36], setting a 30 min seizure occurrence period (SOP) and a 3 min seizure prediction horizon (SPH), as illustrated in Figure 4. The SOP refers to a critical period during which epilepsy patients are at potential risk of experiencing a seizure, while the SPH describes the time span from when the system issues an imminent warning of entering the SOP to the actual occurrence of a seizure. If the model successfully predicted a seizure during the SOP, such a prediction was considered a true positive, indicating the model’s accuracy. Conversely, if the prediction system indicated an impending seizure during the SOP, but no seizure actually occurred, this was considered a false alarm.

A rigorous evaluation method was used to assess the performance of the proposed seizure prediction model by introducing a confusion matrix as an evaluation tool. These metrics included accuracy, sensitivity, FPR, F1 score, and AUC. The formulas are calculated as follows:

(5) $$Accuracy=\frac{TP+TN}{TP+TN+FP+FN}$$

(6) $$Sensitivity=\frac{TP}{TP+FN}$$

(7) $$F1‐score=\frac{2TP}{2TP+FP+FN}$$

Here, *TP* is true positive, *TN* is true negative, *FP* is false positive, and *FN* is false negative. FPR denotes the number of events per hour that the continuous period of the inter-ictal period was misjudged as the pre-ictal period.

Cross-validation is a commonly used technique for evaluating the effectiveness of statistical methods by examining their applicability to independent datasets [37]. Our experiments were conducted using a patient-specific setting and leave-one-out cross-validation (LOOCV) methods. Specifically, assuming a patient had *N* seizures, the pre-ictal EEG signals were divided into *N* parts in sequence; the (*N* − 1) parts were used for training models while the remaining one was selected for testing verification. The same processing was performed on the inter-ictal EEG data. *N* iterations were performed and the average metric results for each patient were reported.

**Figure 5:**
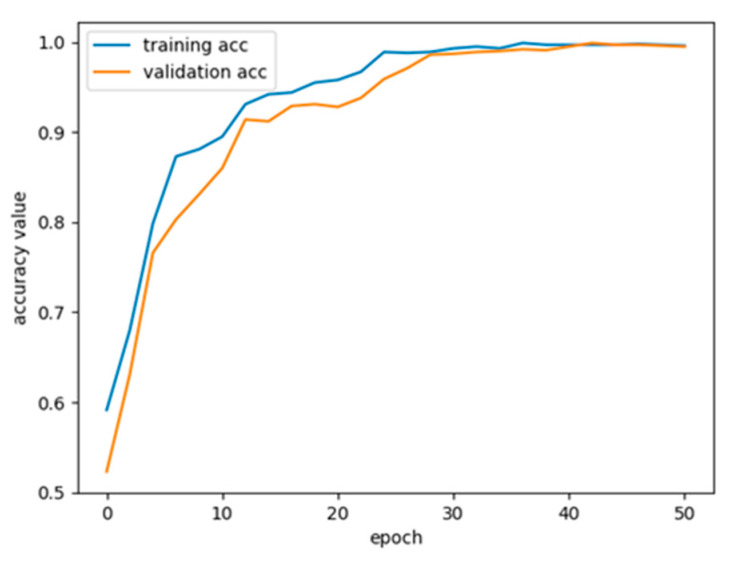
The training and validation accuracy curve of the proposed model for the CHB01 patient.

Table 2 presents the performance data for all patients. The classification accuracy was assessed in 24 instances, leading to a mean accuracy of 93.65%. Apart from CHB13, CHB16, and CHB22, the remaining instances exhibited a classification accuracy exceeding 90%. Twelve patients had a false positive rate below 0.1/h, and only one patient had a false positive rate above 0.2/h. Figure 5 illustrates the accuracy curve for the CHB01 case during training. The accuracy of the training set increases rapidly in the first 20 epochs, then gradually levels off, achieving a stable state between 30 and 40 epochs.

### 3.3. Ablation Experiment

To illustrate the effectiveness of our proposed DenseNet–ViT hybrid model and the individual contributions of each component, we carried out ablation experiments, which serve as benchmarks to gauge the performance of our hybrid model.

The ablation experiments were as follows:

(1) Standalone DenseNet model: A standalone DenseNet model was trained and evaluated on the preprocessed EEG dataset. This experiment enabled us to assess DenseNet’s effectiveness in extracting spatial features and its contribution to the hybrid model.
(2) Standalone ViT model: Similarly, we evaluated an independent ViT model on the same dataset. This experiment determined the effectiveness of ViT in capturing long-range dependencies and global contextual information.

**Figure 6:**
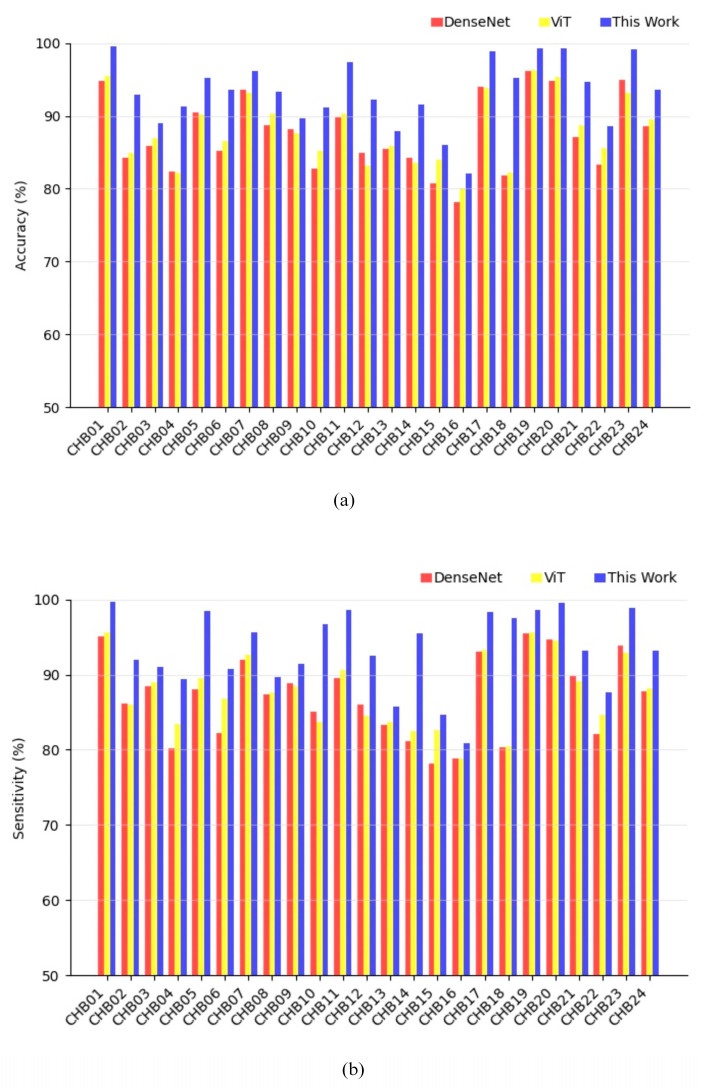
Comparison of the accuracy and sensitivity results of DenseNet, ViT, and hybrid models of this work for each patient. (**a**) The accuracy results and (**b**) the sensitivity results.

Figure 6 demonstrates the comparison of accuracy and sensitivity results for each patient between the proposed method and the control group. The standalone DenseNet model achieved an average accuracy of 87.52%, sensitivity of 86.98%, F1 score of 0.867, AUC of 0.877, and FPR of 0.124/h. These outcomes emphasize the ability of DenseNet to effectively manage spatial features in the data and accomplish a satisfactory performance. In contrast, the ViT model scored an average accuracy of 88.1%, a sensitivity of 87.68%, an F1 score of 0.88, an AUC of 0.888, and a lower FPR of 0.105/h. These results suggest that ViT is capable of handling long-range and global contextual information. By combining the strengths of both architectures, our proposed DenseNet–ViT hybrid model can potentially achieve superior performance.

## 4. Discussion

In this study, a novel method for predicting seizures is proposed by integrating DenseNet and ViT models, which marks the first exploration into seizure prediction methods using this combination. Enhanced performance is attributed to the effective integration of spatial and temporal features extracted from the EEG data, which allowed the model to capture more comprehensive and discriminative information. Moreover, the attention-based fusion layer played a crucial role in weighing and combining the feature representations from the two individual models.

**Figure 7:**
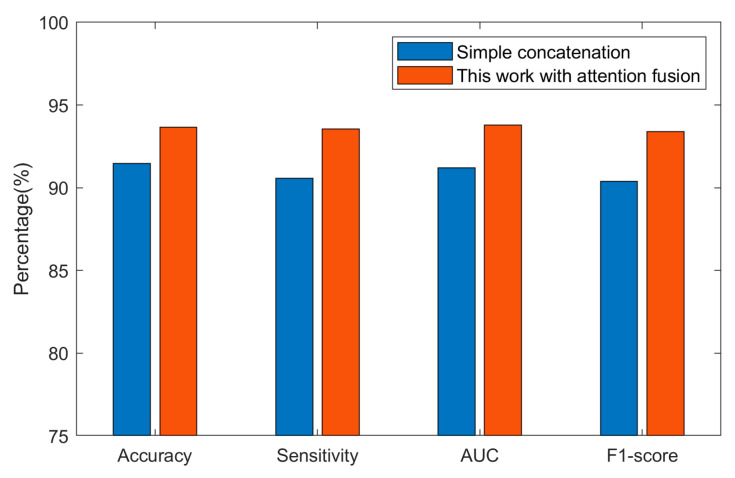
Comparison of the proposed hybrid model with attention fusion and simple concatenation without attention fusion.

To investigate the role of the attention-based fusion layer in our DenseNet–ViT hybrid model, we also conducted a study using alternative fusion strategy. In this experiment, we replaced the attention-based fusion layer with a simple concatenation approach. That is, we directly concatenated the output feature maps from both the DenseNet and ViT components and fed them into the original classification head of the ViT model to generate the final prediction. Figure 7 shows the comparison results of the hybrid DenseNet–ViT model with attention fusion and the simple concatenation without attention fusion.

In the study where the attention-based fusion layer was replaced with simple concatenation, the model attained an average accuracy of 91.46%, sensitivity of 90.56%, F1 score of 0.904, AUC of 0.912, and FPR of 0.093/h. These results substantiate that even without the attention mechanism, the model can maintain commendable performance, showcasing the inherent strength of the DenseNet and ViT components in seizure prediction. However, the performance metrics were lower than those achieved with the attention-based fusion layer. This difference in performance illuminates the crucial role that the attention mechanism plays in the hybrid model. The attention-based fusion layer is designed to carefully weigh and fuse the features extracted by DenseNet and ViT, leveraging the interdependencies and saliency of each feature to optimize their contribution to the seizure prediction task. In contrast, simple concatenation, although effective, treats all features uniformly, disregarding the relevance and importance of individual features for the prediction task.

**Figure 8:**
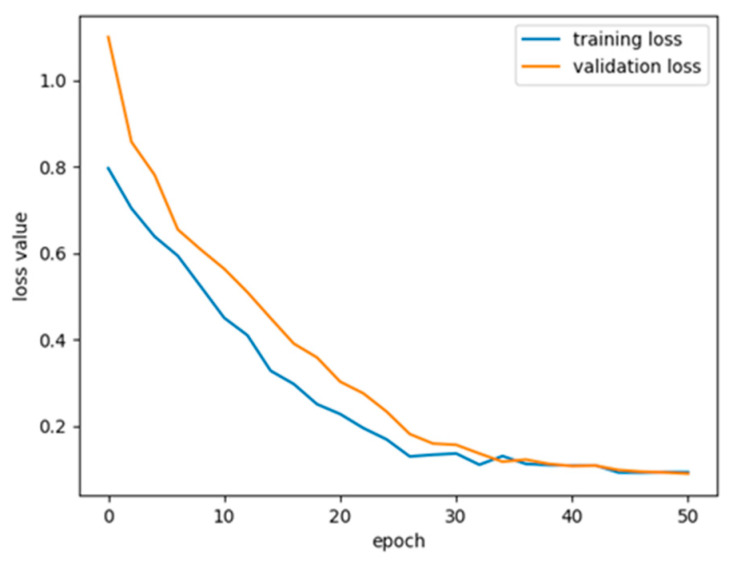
The training and validation loss value curves of the proposed model for the CHB01.

In addition, the overfitting problem in deep learning network models cannot be ignored. In this study, the Adam optimization algorithm was combined with the cosine annealing strategy to effectively prevent the overfitting of training data and enhance the generalization ability of models for unknown data by reducing the oscillation of the learning rate and periodic restart. The improved algorithm used an adaptive learning rate mechanism to effectively handle the complex features of EEG data, especially in hybrid models. In our model, the cross-entropy loss function was used, the core concept of which is to “penalize” the model for incorrect predictions of actual events. As illustrated in Figure 8 for the CHB01 case, both the training loss and the validation loss significantly decrease with each epoch at the early stage, indicating continuous improvement in the model during the learning process. As training continues, both curves tend to flatten and gradually converge, indicating that the model is converging.

Furthermore, the early stopping technique, a technique particularly suitable for training deep learning networks, was applied in this experiment. Its core principle is to terminate training prematurely once the model’s performance on the validation set no longer shows significant improvement or begins to decline, thereby preventing overfitting. This strategy not only quickly identifies optimal model parameters, but also serves as an effective regularization technique to simplify model complexity. After multiple trials, we set the patience value of early stopping to six, meaning training would be prematurely terminated if there were no improvements in the performance on the validation set over six consecutive training epochs. This approach avoids premature stopping before the model has fully converged and saves unnecessary computational resources and time.

The application of CNN as a classifier for seizure prediction constitutes a classic approach, achieving notable success. Khan et al. achieved a sensitivity of 87.8% through the utilization of wavelet transform preprocessing, subsequently using CNN for categorization [38]. Zhang et al. presented a distinctive approach for anticipating epileptic seizures, which merges common spatial patterns (CSP) and CNN. This method attained a sensitivity of 92.2% and a false prediction rate of 0.12 per hour [39]. Ozcan et al. utilized spectral band power, statistical moments, and Hjorth parameters as feature inputs for 3D CNN classification, reaching a sensitivity of 85.7% [40]. However, the pure CNN approach exhibits certain limitations. CNNs primarily focus on spatial information for feature extraction and modeling, potentially neglecting long-term dependencies and dynamic information in time series data. Consequently, integrating CNNs with other types of neural networks, such as RNNs (e.g., LSTM or GRU) or transformer networks, may be considered. These hybrid approaches could be more adept at capturing temporal information and channel correlations in EEG signals, thus enhancing epilepsy prediction performance.

The success of ViT in the field of computer vision has inspired researchers to extend its application to EEG data processing. Although the application of VIT in this domain is still in its early stages of research, it has demonstrated promising potential. Zhang et al. filtered the original EEG signals of patients, preprocessed the filtered signals using the STFT, and finally classified them with the VIT model, obtaining an accuracy of 81.19% and sensitivity of 75.58% [25]. Godoy et al. developed a transformer-based model, a temporal multi-channel vision transformer (TMC-ViT), for multi-channel temporal signals and validated it on the CHB-MIT dataset, obtaining an accuracy of 82% [41]. Recently, Deng et al. suggested an innovative hybrid visual transformer (HViT) structure, which employed CNN to enhance the capacity of transformers for handling local features. They achieved the top sensitivity of 87.9%, a FPR of 0.056/h, and an AUC of 0.937 [27]. Compared with their method, the hybrid DenseNet–ViT model proposed in this study has a higher sensitivity with similar AUC values and a slightly higher FPR, which indicates that our model has better recognition performance for pre-ictal EEG data. In addition, we used all patient data in the dataset, while they used part of the EEG dataset. Table 3 displays a comparison of our method’s performance with that of other suggested techniques.

The results of this study reveal how to effectively utilize DenseNet and ViT networks to perform feature learning on multi-channel EEG data simultaneously. The DenseNet module effectively extracts spatial information in EEG data, while the ViT module enhances the capture of long-term dependencies and global information. The feature fusion layer based on the attention mechanism effectively combines the advantages of both. The results showed that the prediction accuracy of models for seizures was improved, demonstrating its potential clinical application value as an EEG-based epileptic seizure early warning system. The hybrid method proposed in this article can accurately and quickly predict future epileptic seizures, provide patients with the opportunity to take quick-acting drugs and safety measures during epilepsy-prone periods, and establish a closed-loop epilepsy intervention system to abort impending epileptic seizures. In addition, the model adopts a patient-specific training method, there is no overlap in EEG data between different patients, and the privacy of data can be guaranteed to a certain extent when used in clinical applications.

However, this research still has certain limitations. Firstly, this study was only conducted and evaluated on the CHB-MIT scalp EEG dataset. Although from the results obtained, the method proposed in this study improves the performance of predicting seizures to some extent, we would attempt to test it on other EEG datasets to obtain more general comparative results to verify its robustness. At the same time, we should also further explore other neural network architectures and investigate more techniques, such as the domain adaptation technique, to probe the model’s interpretability and robustness in subsequent work.

Secondly, given the complexity of the hybrid depth model, there are still some challenges in making it applicable in clinical settings, so we would further attempt to improve and streamline the network architecture to better apply it to clinical seizure prediction. In this study, the proposed model was conducted in patient-specific scenarios, and we will also focus on the execution of cross-patient experiments, which could better explore the model performance in different patient groups. In addition, combining EEG signals with additional data modalities, such as fMRI or MEG, to explore epileptic seizure prediction is also a meaningful future direction.

## 5. Conclusions

In this research, a unique deep learning-based hybrid model for epileptic seizure prediction was developed by merging DenseNet and ViT architectures. The suggested model effectively integrated spatial and temporal features derived from EEG data, enabling it to attain superior predictive performance compared to standalone DenseNet and ViT models, as well as other individual models and benchmark algorithms. The attention-based fusion layer played a pivotal role in the model’s effectiveness, showcasing the potential of combining the strengths of different deep learning models in the epilepsy research domain. Future research should focus on refining the proposed model, exploring more sophisticated fusion strategies, and incorporating additional patient-related information to enhance predictive capabilities.

## Figures and Tables

**Table 1:** Details of the CHB-MIT dataset used in this study.

Case	Gender	Age	Record Time (h)	Duration of Seizures (s)	Number of Seizures (No Merge)	Number of Seizures ^#^ (Merge)
CHB01	Female	11	40.6	442	7	7
CHB02	Male	11	35.3	172	3	3
CHB03	Female	14	38.0	402	7	7
CHB04	Male	22	155.9	382	4	4
CHB05	Female	7	39.0	558	5	5
CHB06	Female	1.5	66.7	147	10	10
CHB07	Female	14.5	67.0	328	3	3
CHB08	Male	3.5	20.0	919	5	5
CHB09	Female	10	67.8	276	4	4
CHB10	Male	3	50.0	454	7	7
CHB11	Female	12	34.8	806	3	2
CHB12	Female	2	20.6	1487	40	21
CHB13	Female	3	33.0	547	12	10
CHB14	Female	9	26.0	169	8	8
CHB15	Male	16	40.0	1992	20	17
CHB16	Female	7	19.0	84	10	9
CHB17	Female	12	21.0	293	3	3
CHB18	Female	18	35.6	317	6	6
CHB19	Female	19	30.0	236	3	3
CHB20	Female	6	27.5	294	8	8
CHB21	Female	13	33.0	199	4	4
CHB22	Female	9	31.0	204	3	3
CHB23	Female	6	26.5	424	7	7
CHB24	-	-	21.3	511	16	14
Total	-	-	979.6	11,646	198	170

^#^ When two seizures are separated by less than 30 min, they are combined into one seizure.

**Table 2:** The method performance metrics (SOP = 30 min, SPH = 3 min).

Case	Accuracy (%)	Sensitivity (%)	FPR (/h)	AUC	F1-Score
CHB01	99.51	99.67	0.002	0.99	0.99
CHB02	92.88	91.96	0.119	0.92	0.91
CHB03	90.13	90.98	0.151	0.92	0.92
CHB04	91.27	89.42	0.132	0.93	0.92
CHB05	95.20	95.51	0.073	0.96	0.95
CHB06	93.62	90.77	0.041	0.95	0.92
CHB07	96.17	95.66	0.031	0.96	0.94
CHB08	93.32	89.61	0.062	0.93	0.92
CHB09	90.67	91.39	0.139	0.91	0.91
CHB10	91.18	93.65	0.143	0.90	0.92
CHB11	97.31	98.61	0.011	0.96	0.95
CHB12	92.17	92.56	0.130	0.91	0.93
CHB13	87.88	85.71	0.147	0.90	0.89
CHB14	91.62	95.50	0.138	0.93	0.95
CHB15	90.97	89.64	0.152	0.92	0.90
CHB16	85.11	87.81	0.201	0.89	0.88
CHB17	98.90	98.31	0.006	0.97	0.98
CHB18	95.16	97.52	0.017	0.95	0.97
CHB19	99.28	98.66	0.003	0.99	0.99
CHB20	99.33	99.56	0.003	0.99	0.99
CHB21	94.66	93.18	0.108	0.94	0.93
CHB22	88.56	87.69	0.120	0.89	0.88
CHB23	99.13	98.92	0.012	0.99	0.97
CHB24	93.58	93.16	0.047	0.92	0.92
Average	93.65	93.56	0.084	0.938	0.934

**Table 3:** Performance comparison on the CHB-MIT dataset for different methods.

Authors	Year	Case of Patients	Method	Acc (%)	Sen (%)	FPR (/h)	AUC	F1-Score
Khan et al.	2017	13	CWT + CNN	-	87.8	0.147	-	-
Truong et al.	2018	13	STFT + CNN	-	81.2	0.16	-	-
Tsiouris et al.	2018	24	LSTM	-	99.2	0.11	-	-
Zhang et al.	2019	23	wavelet packet + CNN	90.0	92.0	0.12	0.90	0.91
Ozcan et al.	2019	16	3D CNN	-	85.71	0.096	-	-
Bhattacharya et al.	2021	21	STFT + Transformer	-	98.46	0.124	-	-
Zhang et al.	2022	14	STFT + ViT	81.19	75.58	-	0.85	-
Godoy et al.	2022	22	TMC-ViT	82.0	80.0	-	0.89	-
Deng et al.	2023	18	HViT-DUL	-	87.9	0.056	0.937	-
This work	2024	24	STFT + DenseNet–ViT	93.65	93.56	0.083	0.938	0.934

## Data Availability

The original CHB-MIT scalp EEG dataset presented in the study is openly available at the website https://archive.physionet.org/pn6/chbmit/ (accessed on 18 August 2024).
